# Triboelectric Energy Harvesting of the Superhydrophobic Coating from Dropping Water

**DOI:** 10.3390/polym12091936

**Published:** 2020-08-27

**Authors:** Jiaxuan Niu, Wenjie Xu, Kaiyi Tian, Gang He, Zhengyong Huang, Qiang Wang

**Affiliations:** State Key Laboratory of Power Transmission Equipment & System Security and New Technology, School of Electrical Engineering, Chongqing University, No. 174 Shazhengjie, District Shaping, Chongqing 400044, China; 20173420@cqu.edu.cn (J.N.); xuwenjie@cqu.edu.cn (W.X.); 20173458@cqu.edu.cn (K.T.); hegang@cqu.edu.cn (G.H.); wangqiang0609@cqu.edu.cn (Q.W.)

**Keywords:** superhydrophobic, mechanical durability, energy harvesting

## Abstract

In this paper, the superhydrophobic coating was prepared by spraying the composites of fluorocarbon emulsion and nanosized silica on the conductive glass sheet for the triboelectric energy harvesting from water droplets. The low surface energy of fluorine in the fluorocarbon emulsion and nanosilica renders the coating with the static contact angle and sliding angle of 156.2° and 6.74°, respectively. The conductive aluminum tape was attached on the surface of the superhydrophobic coating to complete the circuit constituted with the aluminum electrode, charged superhydrophobic coating, and the conductive glass sheet. During the contact electrification with the bouncing water droplet, the superhydrophobic coating with the aluminum electrode can obtain the electric energy with an open-circuit voltage of 20 V and short-circuit current of 4.5 μA, respectively. While the control device only produced an open-circuit voltage of 0.2 V. The generated power by one drop was enough to light up 16 commercial LEDs. Results demonstrate that the fluorocarbon/silica composite superhydrophobic coating is potentially a strong candidate for scavenging energy in sliding mode from raindrops.

## 1. Introduction

The superhydrophobic coating demonstrates extreme hydrophobicity with a static angle greater than 150°, sliding angle lower than 5°. Water droplets on the superhydrophobic coating keep almost spherical, significantly reducing the contact area and adhesion force between the superhydrophobic surface and water droplets [1,2]. Therefore, superhydrophobic coatings are widely used in self-cleaning [3,4], oil-water separation [5,6], anti-icing [7,8], leakage current suppression [9], and other aspects.

Nowadays, it is found that the electrostatic induction of the tribo-charges on the water/solid interface on the hydrophobic coating has emerged as an effective technology for the water-related energy harvesting [10,11,12]. B. Ravelo et al. demonstrate the triboelectricity by the flow of water in an insulating pipe, a voltage variation up to 300 mV is observed by using pipes having mm-diameter and pure water with 45 cm^3^/s flow rate [13]. Dongwhi Choi et al. report that a droplet dispensed from a micropipette almost always has a considerable electrical charge of a magnitude. The surface charge density of 4.5 μC/m^2^ is measured on each water droplet pipetted from a polytetrafluoroethylene (PTFE) tip [14]. The surface of the superhydrophobic coating has abundant hierarchical micronano structures. The structures on the friction surface are able to significantly increase the contact area. At the same time, the rapid separation of droplets after collision with the superhydrophobic surface could potentially improve the output performance of energy harvester. 

The energy harvester with superhydrophobic coating adopted liquid itself as a triboelectric material, and show the spotlight to overcome the inevitable friction wear between two solid materials in conventional TENGs (triboelectric nanogenerator) [15,16]. Jihoon Chung et al. developed a sprayed-on TENG using a commercial hydrophobic spray that can easily create a superhydrophobic surface. The electrical output depends on the number of topcoats applied on the solid surface. The sprayed-on superhydrophobic surface produced an average positive peak V_OC_ of 13.4 V and I_CC_ of 2.1 µA under continuous water sprinkling from a commercial showerhead [17]. Jeong Hwan Lee et al. reported the development of contact electrification-based water droplet-driven triboelectric nanogenerator for harvesting energy from the water-droplet bouncing between two superhydrophobic surfaces. The device produced a short-circuit current and an open-circuit voltage of 1.3 μA and 1.4 V, respectively [18]. Wanghuai Xu et al. developed a device to harvest energy from impinging water droplets by using an architecture that comprises a polytetrafluoroethylene film on an indium tin oxide substrate plus an aluminum electrode. An individual DEG (drop electricity generator) indicates that the open-circuit output voltage and short-circuit current were about 143.5 V and 270.0 μA [19]. However, the investigation of the demonstration of the water contact electrification of the superhydrophobic coating-based energy harvester with a closed-loop electrical circuit is seldom reported.

In this paper, the superhydrophobic coating was fabricated by spraying fluorocarbon emulsion and nanosilica composite on the glass plate. The microstructures and mechanical performances of the superhydrophobic coating were evaluated. The static contact angle and sliding angle of the coating are 156° and 6.74°, respectively. The durable coating demonstrates energy harvesting capability from the water contact electrification. The single-electrode TENG generated an open-circuit voltage of 0.2 V. And the superhydrophobic coating based energy harvester with an aluminum electrode produced an open-circuit voltage of 20 V and a short-circuit current of 4.5 μA, which could directly light up 16 commercial LEDs.

## 2. Materials and Methods

### 2.1. Materials

The fluorocarbon emulsion(FC-3150) was purchased from Solmont Technology Co., Ltd. (Wuxi, China). Tetraethyl orthosilicate (TEOS) was obtain from Changzhou Zhongjie Chemical Co., Ltd. (Guangzhou, China). 1H,1H,2H,2H-perfluorodecyltrimethoxysilane (FAS-17, 97 wt %) was got from Aladdin Co., Ltd. (Shanghai, China). The aminopropyl triethoxysilane (KH550), and FAS-17 were supplied by Saiwo chemical Co., Ltd. (Wuhan, China). All the reagents were analytically pure.

### 2.2. Surface Modification of SiO_2_

The surface hydrophobicity and the surface roughness can be both improved by adding fluorine modified silica nanoparticles. The hydrophilic silicon dioxide is difficult to be dispersed in the organic solvent and tends to aggregate through hydrogen-bond interaction and electrostatic interaction [20,21]. It is necessary to modify the surface of silicon dioxide to improve the compatibility with fluorocarbon resin and the base dispersity, and the modification process is mainly divided into two steps.

Firstly, 300 mL of tetraethyl orthosilicate was dissolved in 3000 mL of anhydrous ethanol to obtain a mixture of anhydrous ethanol and TEOS. Then the mixture above was poured into a 5000 mL three-mouth flask equipped with the electric mixer, a drip funnel, and a condenser. Eight wt % of the aminopropyl triethoxysilane (KH550) dissolved in the ethanol was loaded into the dropping funnel. The switch of the dropping funnel was opened and adjusted to start the agitator. The rate of dropping the KH550 solution into the flask was controlled to be 15 mL/min.

**Figure 1:**
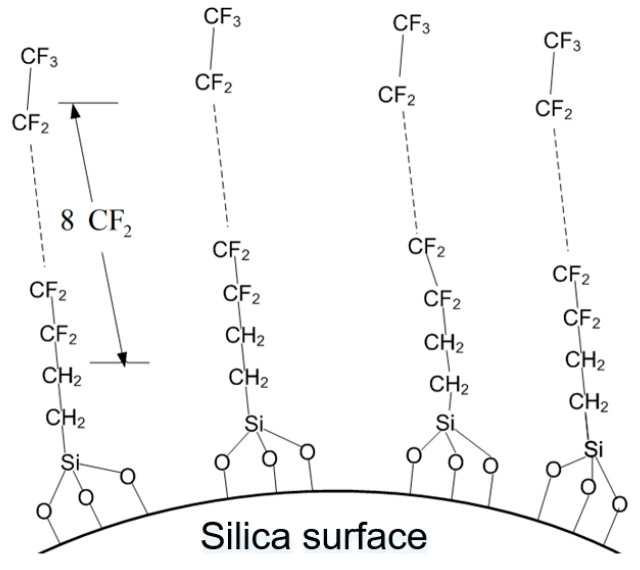
Schematic diagram of the surface modification of nanosilica with FAS-17 (1H,1H,2H,2H-perfluorodecyltrimethoxysilane).

The surface of the prepared silicon dioxide sol was treated with FAS-17. Forty ml of FAS-17 solution (2 wt % in the ethanol) was added into a three-necked flask containing the nanosilica sol obtained in the previous step, and about 0.3 mL of acetic acid was used for acidification. Then the agitator was opened for stirring. Next, the reactant was treated in a water bath at 60 °C for 6 h. Finally, the hybrid sol was dried in a vacuum drying oven with 100 °C for 12 h. The surface modification of nanosilica with FAS-17 was obtained after grinding, and is schematically shown in Figure 1.

### 2.3. Preparation of Superhydrophobic Coating

Fluorocarbon emulsion and modified silica particles were mixed to improve the superhydrophobicity of the surface. When the concentration of SiO_2_ particles is small, there are not enough nanoparticles to form a complete micronano structure on the surface of the coating. The optimum concentration of silica particles in the experiment is around 8 wt %. The modified nanosilica with a weight of 6 g was added to the ethanol(150 mL, 75 wt %) and ultrasonically dispersed for 5 min under room temperature. Then, fluorocarbon emulsion was added into the mixture, which was stirred thoroughly, and then dispersed by ultrasound for another half an hour to obtain the painting mixture. The sample placed in the container equipped with a high-pressure spray gun was sprayed evenly on a clean conductive glass plate. A superhydrophobic coating made of fluorocarbon resin and modified nanosilica was obtained after being thoroughly dried.

### 2.4. Fabrication of the Triboelectric Energy Harvester

The purchased indium tin oxide (ITO) slide was ultrasonically cleaned in acetone for 10 min. After blown dried with nitrogen, the slide was immersed in a fluorocarbon mixture, dried under room temperature, and the ITO was coated with the fluorocarbon/silica superhydrophobic coating. Then, an aluminum electrode was adhered on the superhydrophobic surface. Finally, the conducting wire was used to connected the ITO and aluminum electrode for subsequent measurements.

### 2.5. Characterizations

Hydrophobicity of the superhydrophobic coating was measured by a contact angle measuring instrument (Drop Meter A-20, Maishi, Ningbo, China). Five different points on the coating were measured to obtain the static contact angles of water droplets on the coating. The slip angle of the superhydrophobic surface was confirmed when the water droplet just started to slide on the measuring platform. The microstructure of the superhydrophobic fluorocarbon/nanosilica was analyzed by field emission scanning electron microscope (FE-SEM, Zeiss Technology Co., Ltd., Suzhou, China). The chemical compositions of the coating were characterized by attenuated total reflection Fourier-transform infrared spectroscopy (ATR-FTIR, Bruker Technology Co., Ltd., Beijing, China). The hardness of the as-prepared superhydrophobic coating was determined by a pencil hardness tester (Bohui Instrument and Meter Co., Ltd., Xi’an, China). The cross-cut tester (Jieke Automation Equipment Co., Ltd., Dongguan, China) is used to determine the adhesion of fluorocarbon superhydrophobic coatings.

During the energy harvesting testing, the dropping water was applied to the superhydrophobic surface for the measurement of typical electrical output. A Keithley 6514 (Keithley Instruments, Inc., Johnston, OH, USA) was used to test the electrical outputs of the coating. The entire test was carried out in an ambient environment. The triboelectric performance, including open-circuit voltage (V_oc_) and short-circuit current (I_sc_) were measured by a Keithley 6514. The electrodes of the energy harvester with the superhydrophobic coating were connected to the output port and ground.

## 3. Results and Discussion

### Surface Hydrophobicity

**Figure 2:**
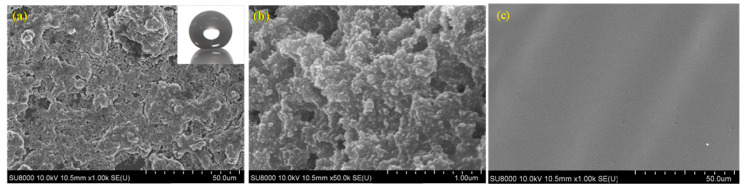
FE-SEM (field emission scanning electron microscope) images of the fluorocarbon superhydrophobic surface (**a**) with the magnification of 1000 times; (**b**) with the magnification of 50,000 times; (**c**) coatings without modified SiO_2_.

The microstructure of nanosilica-doped fluorocarbon superhydrophobic was analyzed by field emission scanning electron microscope (FE-SEM), and the results are shown in Figure 2. It can be seen from the FE-SEM images that irregular micron-scale protrusions are distributed on the surface. The protrusion sizes ranged between 10 μm and 24 μm. The gap with an interval of 20 microns around is formed between the protrusions. With the magnification of 50,000 times, lots of sub-micron-level protrusions are observed on the micro-scale surface of the superhydrophobic surface. Besides, nano-scale protrusions with sizes of 40 nm around are observed in the surface of the micro-scale protrusions. As shown in Figure 2c, the surface of the coating without modified nanosilica is quite smooth.

By comparing the SEM images of the coatings at different magnifications, the surface of the nano-modified fluorocarbon superhydrophobic coating has a dual micronano-scale composite rough structure. The nanoparticles were evenly distributed on the surface of the coating without apparent agglomeration. After the thermal curing, the coating with no cracking demonstrates great superhydrophobicity.

**Figure 3:**
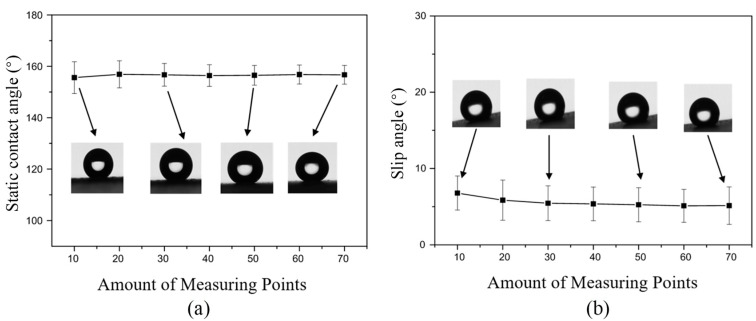
Water morphology on the fluorocarbon superhydrophobic coating (**a**) the static water contact angle of water droplets; (**b**) the slip angle of water droplets.

As shown in Figure 3a, the static contact angle of the as-prepared superhydrophobic coating was analyzed and maintained between 153° and 163°, with an average value of 156°. According to Equation (1), *θ_C_* is the Cassie-Baxter apparent contact angle of the superhydrophobic surface, that is, the measured degree of 156.2°. *f_SL_* and *f_LV_* are the fractional surface area of solid/liquid and gas/liquid interface, respectively. *θ_S_* is the solid-liquid contact angle of the fluorocarbon resin, which was measured to be 110°. *θ_V_* is the vapor-liquid contact angle considered to be 180°. Therefore, it can be calculated that the ratio of the solid-liquid contact surface (*f_SL_*) in the contact interface of the water droplet and the superhydrophobic coating is 12.9%, and the ratio of the gas-liquid contact surface (*f_LV_*) is 87.1%. Result shows that the actual contact area between the superhydrophobic coating and water droplets only accounts for 12.9% of the entire composite contact surface area, while the contact surface area occupied by air is 87.1%. Importantly, the result indicates that the fluorocarbon superhydrophobic coating has a high static contact angle and can achieve superhydrophobicity.

(1) $$\cos\theta_{c}=f_{SL}\cos\theta_{s}+f_{LV}\cos\theta_{V}$$

The average slip angle of the sample was 6.74° as shown in Figure 3b, which indicates that the surface of the as-prepared superhydrophobic coating has little drag effect on water droplets. This excellent hydrophobicity is conducive to the free movement of water droplets and can improve the contact separation performance for energy harvesting.

**Figure 4:**
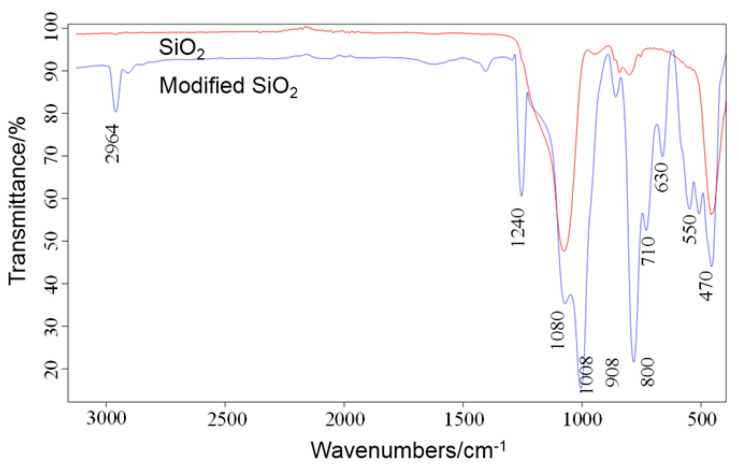
ATR-FTIR (attenuated total reflection Fourier-transform infrared spectroscopy) image of SiO_2_ and modified SiO_2_ particles.

Figure 4 shows the FTIR spectrum of nanosilica modified FC-3150 superhydrophobic coating. It can be seen from the figure that there are only strong absorption peaks at 1080 cm^−1^ and 470 cm^−1^ on the FTIR spectrum line of nanosilica particles, which correspond to the stretching vibration and bending vibration of nanosilica Si-O group, respectively. On the ATR-FTIR spectrum line of SiO_2_ and modified SiO_2_ particles, absorption peaks were observed at 2964 cm^−1^, 1240 cm^−1^, 1080 cm^−1^, 1008 cm^−1^, 908 cm^−1^, 800 cm^−1^, 710 cm^−1^, 630 cm^−1^, 550 cm^−1^ and 470 cm^−1^. Among them, absorption peaks at 1355 cm^−1^ and 1240 cm^−1^ correspond to the stretching vibration of the C-F groups on the FAS-17 molecular chain. The absorption peak at 710 cm^−1^ corresponds to the stretching vibration of the C-C group on the long chain of FAS-17 molecule, indicating that the C-F group has completed the modification of nanosilica particles with low surface energy. The absorption peaks at 630 cm^−1^ and 550 cm^−1^ correspond to the stretching and bending vibration of the Si-C group. Because of the quite low surface energy of the C-F group, and the micronano binary rough structure combined on the surface of the coating, the coating has excellent superhydrophobicity. The fluorine groups have been grafted on the surface of the silica particles, which was combined in the superhydrophobic coating.

The hardness of the coating demonstrates the mechanical strength of the surface, which can reflect the tensile and compressive strength of the coating surface. In the long-run application in terms of surface resistance or sliding friction, the coating surface will be affected by mechanical impact, raining, weathering, etc. The coating surface must have a certain mechanical hardness to maintain a long service life. Therefore, the hardness of the as-prepared superhydrophobic coating should be determined

A layer of fluorocarbon superhydrophobic coating was applied on the glass testing board. After it was completely dried, the coating sample was fixed horizontally. The fixed pencils with different hardnesses were advanced on the coating surface with an angle of approximately 45°, at a speed of 1 mm/s. Scratch with length around 3 mm was formed on the coating surface with mechanical strength enough to scratch the coating. 

**Figure 5:**
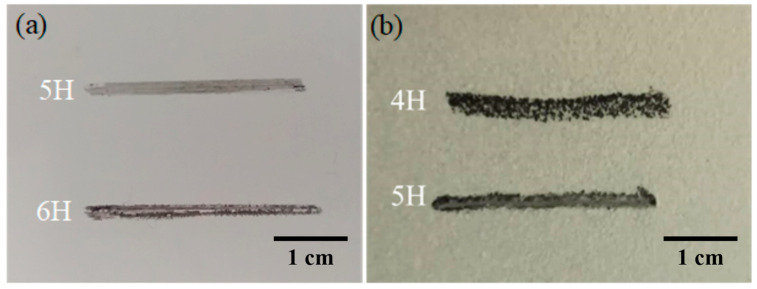
Surface hardness of the superhydrophobic coating. (**a**) Without and (**b**) with modified SiO_2_.

The result in Figure 5a showed that the hardness of the superhydrophobic coating without modified SiO_2_ reached the 6H level. And that of fluorocarbon superhydrophobic coating reached the 5H level, which met the requirements for practical application, as shown in Figure 5b. The bottom scratch shows that the coating keeps undamaged with the test of the hardness of 4H and 5H, respectively.

Film adhesion refers to the firmness between the coating and the substrate, which is one of the basic standards to measure the long-term stability of the coating. A layer of fluorocarbon superhydrophobic coating was applied evenly on the test board, and three testing positions were randomly selected on the board. The distance between each other is not less than 5 mm.

**Figure 6:**
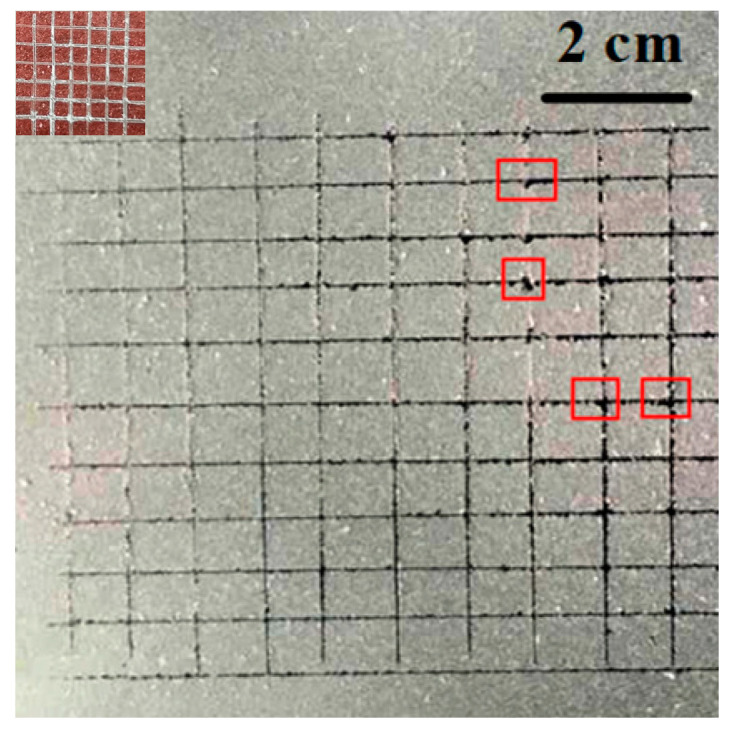
Adhesion testing of fluorocarbon superhydrophobic coating.

The blade is perpendicular to the surface of the sample with uniform force, cutting at a speed of 20–50 mm/s and penetrating through the substrate. If the coating is too thick or hard to be penetrated, the test is invalid. The adhesion is measured as the percentage of the complete square on the coating surface. As shown in Figure 6, the 100-grid testing was performed on the fluorocarbon superhydrophobic coating. The results showed that the cut on the edge of the grid on the coating surface had no obvious peeling marks, and the grid remained intact without separation. There are a few pieces of the coating that is peeling at four junctions of the incisions highlighted in the red boxes. Thus, the adhesion of the as-prepared superhydrophobic coating on the glass substrate is grade 1, according to the adhesion grade standard of Paints and varnishes-Cross cut test for films (GB/T9286-88). The inset of Figure 6 showed the adhesion of the coating without nanosilica. There was almost no peeling on the edge of the grid on the coating surface, indicating that the adhesion of the coating without modified nanosilica is grade 0. With the modification of the nanosilica with KH550, the interface compatibility between SiO_2_ particles and fluorocarbon resin is greatly improved. Nanosilica is formed independently, which enables it to have a strong adhesion with the substrate. 

The triboelectric performance of the superhydrophobic coating was tested by using the falling water from a dropper. Conductive aluminum tape is attached to the ITO slide coated with the fluorocarbon/silica superhydrophobic coating. The conducting wire connected the slide and the aluminum electrode for the electrical test with a Keithley 6514. Then, water was dropped onto the superhydrophobic surface, and the distance between the superhydrophobic surface and the water dropper was around 10 cm. The impact angle was optimized at 30°.

**Figure 7:**
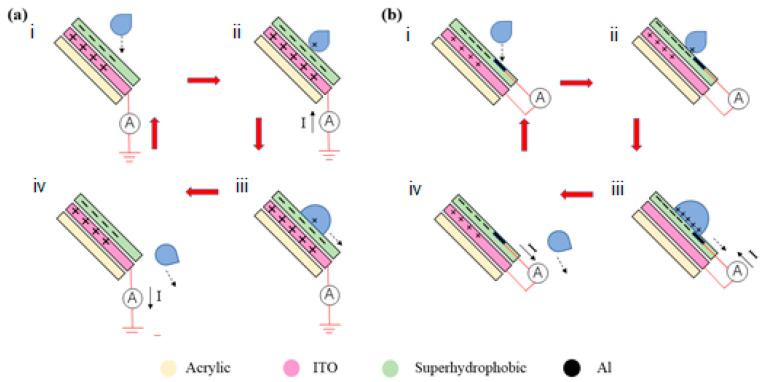
The working mechanism of energy harvesting with superhydrophobic coating. (**a**) Without and (**b**) with an aluminum electrode. i. The droplet falls towards the fluorocarbon/silica superhydrophobic coating. ii. The droplet contacts with superhydrophobic coating. iii. Droplet spreads on the surface and contacts with the aluminum electrode. iv. The droplet leaves the surface.

Figure 7 shows the working mechanism of energy harvesting with the fluorocarbon/silica superhydrophobic coating in different modes. A tap water droplet falls towards the superhydrophobic coating, which stored some negative charges through the previous contact, at the height of 10 cm. The amount of positive charges on the ITO slide is the same due to electrostatic induction. Then the droplet contacts with superhydrophobic coating and a small amount of charge are induced on the superhydrophobic surface as a result of electrification. In the single-electrode mode in Figure 7a, the amount of transferred charge between ITO and the ground is the charge generated by friction between the water and the superhydrophobic coating. While in Figure 7b, it is shown that the droplet is in full contact with superhydrophobic coating and makes contact with the aluminum electrode. Then the ITO, aluminum electrode, and superhydrophobic surface are connected through the droplet. Thus a flow of current between ITO and aluminum electrode is induced. Finally, the droplet leaves the surface, and the ITO and superhydrophobic surface return to the original state.

**Figure 8:**
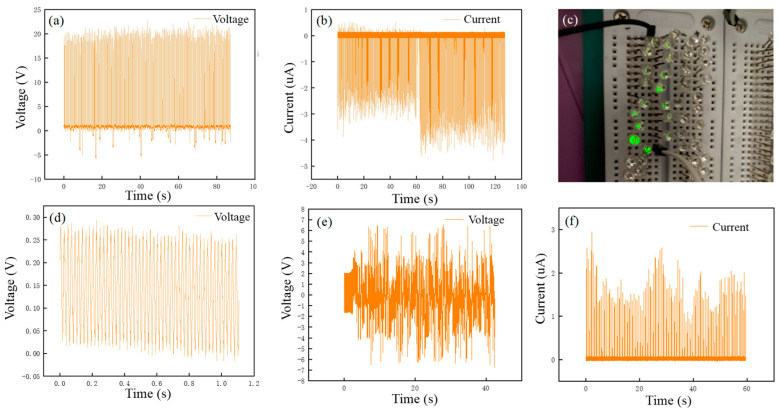
(**a**) Generated Voc from superhydrophobic coating with an aluminum electrode. (**b**) Generated Isc from superhydrophobic coating with an aluminum electrode. (**c**) The image of the LEDs lit up by water droplet. (**d**) Generated Voc from superhydrophobic coating without aluminum electrode. (**e**) Generated Voc from the coating without modified SiO_2_. (**f**) Generated Isc from the coating without modified SiO_2_.

The open-circuit voltage (Voc) and short-circuit current (Isc) of the superhydrophobic coating were measured to evaluate the performance of the energy harvester, as shown in Figure 8. The typical Voc and Isc curves generated from the superhydrophobic coating with an aluminum electrode in Figure 8a,b indicate that the Voc value and Isc achieve 20 V and 4.5 μA, respectively. The Voc of the superhydrophobic coating without aluminum electrode is only 0.2 V, which can be seen in Figure 8d. Figure 8e,f show the triboelectric performance of the coating without modified nanosilica, the Voc value and Isc achieve 7 V and 2 μA, respectively. The electrical output of the fluorocarbon/nanosilica composite superhydrophobic coating measured with a Keithley 6514 is relatively stable during the dripping process.

To better visualize the output power generated by the superhydrophobic TENG, some commercial LEDs were used to test the output of droplets driven TENG. Figure 8c shows that 16 serially connected green LEDs could be directly lit by the TENG that was driven by dropping water at the height of 10 cm. The LEDs constantly flashed at the frequency with which the drop was falling, and the dynamic video can be seen in Supplementary Material Video S1.

## 4. Conclusions

In this paper, a fluorocarbon/nanosilica composite superhydrophobic coating for triboelectric energy harvesting is presented. The fluorocarbon-based nanocoatings demonstrate superhydrophobic performance. The hardness test shows that the superhydrophobic coating meets the requirements for practical applications. The adhesion on the glass substrate is ranked to be grade 1. The durable coating demonstrates potential energy harvesting capability through the liquid-solid surface interaction. The open-circuit voltage of 20 V and a short-circuit current of 4.5 μA generated by the fabricated TENG were obtained. As a comparison, the open-circuit voltage of the single-electrode TENG was measured. Results indicate that the durable superhydrophobic coating shows potential energy harvesting from the water dropping.

## Supplementary Materials

The following are available online at https://www.mdpi.com/2073-4360/12/9/1936/s1, Video S1: The LEDs lit up by the water dropping on the superhydrophobic surface. Figure S1: EDS of the fluorocarbon superhydrophobic coating.
